# 2451. The Impact of Common Variations in SOFA Score Calculations on Sepsis Surveillance Using Sepsis-3 Criteria

**DOI:** 10.1093/ofid/ofad500.2069

**Published:** 2023-11-27

**Authors:** Mohammad Alrawashdeh, Michael Klompas, Chanu Rhee

**Affiliations:** Harvard Medical School, Boston, Massachusetts; Harvard Medical School and Harvard Pilgrim Health Care Institute, Boston, Massachusetts; Brigham and Women's Hospital, Boston, Massachusetts

## Abstract

**Background:**

Sepsis-3 defines sepsis as infection associated with an increase in baseline Sequential Organ Failure Assessment (SOFA) score by ≥2 points. Consistent electronic application of Sepsis-3 criteria could allow for standardized estimates of sepsis incidence and outcomes. However, there are many methods of calculating SOFA scores that may contribute to variability in sepsis surveillance.

**Methods:**

We retrospectively identified all adult patients hospitalized at 5 Massachusetts hospitals between 2015-2022 and calculated the incidence and mortality rates of sepsis, defined as suspected infection (any clinical culture and antibiotics continued for ≥3 days) with concurrent organ dysfunction (increase in SOFA by ≥2 points) using different strategies for calculating respiratory SOFA scores and for handling missing data. We calculated respiratory SOFA scores by 1) using the standard PaO2/FiO2 ratio method, and 2) using SpO2/FiO2 ratios when arterial blood gases were not performed. We managed missing data using: 1) normal value imputation for each organ system if values from that day were missing, 2) mean value imputation, using the mean of the worst values of the preceding and following day, and 3) carry forward imputation, carrying forward the last non-missing observation.

**Results:**

The cohort included 1,064,498 hospitalizations, of which 299,427 (28.1%) had suspected infection. Using the standard respiratory PaO2/FiO2 method, sepsis incidence and mortality rates were 14.2% and 10.1% but incidence rose to 19.7% and mortality dropped to 7.8% using the SpO2/FiO2 respiratory score imputation and normal value imputation. Results were similar with mean value and carry-forward imputation.

**Conclusion:**

We observed substantial differences in calculated sepsis incidence and mortality rates when using SpO2/FiO2 vs PaO2/FiO2 ratios alone to impute respiratory SOFA scores. Common SOFA missing value imputation strategies, however, had little impact on sepsis incidence and mortality rates. These findings highlight an important source of potential variability when applying Sepsis-3 criteria and underscore the need for standardized surveillance methodology.

**Disclosures:**

**Michael Klompas, MD, MPH**, UpToDate, Inc.: Royalties for chapters on pneumonia **Chanu Rhee, MD, MPH**, Cytovale: Advisor/Consultant|Pfizer: Advisor/Consultant|UpToDate, Inc.: Honoraria

